# Cartilage compositional MRI—a narrative review of technical development and clinical applications over the past three decades

**DOI:** 10.1007/s00256-024-04734-z

**Published:** 2024-07-09

**Authors:** Xiaojuan Li, Jeehun Kim, Mingrui Yang, Ahmet H. Ok, Štefan Zbýň, Thomas M. Link, Sharmilar Majumdar, C. Benjamin Ma, Kurt P. Spindler, Carl S. Winalski

**Affiliations:** 1 Program of Advanced Musculoskeletal Imaging (PAMI), Cleveland Clinic, 9500 Euclid Avenue, ND20, Cleveland, OH 44195 USA; 2 Department of Biomedical Engineering, Lerner Research Institute, Cleveland Clinic, Cleveland, OH USA; 3 Department of Diagnostic Radiology, Cleveland Clinic, Cleveland, OH USA; 4 Department of Orthopaedic Surgery, Cleveland Clinic, Cleveland, OH USA; 5 Department of Radiology and Biomedical Imaging, University of California San Francisco (UCSF), San Francisco, CA USA; 6 Department of Orthopaedic Surgery, UCSF, San Francisco, CA USA

**Keywords:** Cartilage, Composition MRI, Quantitative imaging, Biomarkers, Osteoarthritis

## Abstract

Articular cartilage damage and degeneration are among hallmark manifestations of joint injuries and arthritis, classically osteoarthritis. Cartilage compositional MRI (Cart-C MRI), a quantitative technique, which aims to detect early-stage cartilage matrix changes that precede macroscopic alterations, began development in the 1990s. However, despite the significant advancements over the past three decades, Cart-C MRI remains predominantly a research tool, hindered by various technical and clinical hurdles. This paper will review the technical evolution of Cart-C MRI, delve into its clinical applications, and conclude by identifying the existing gaps and challenges that need to be addressed to enable even broader clinical application of Cart-C MRI.

## Introduction

**Fig. 1:**
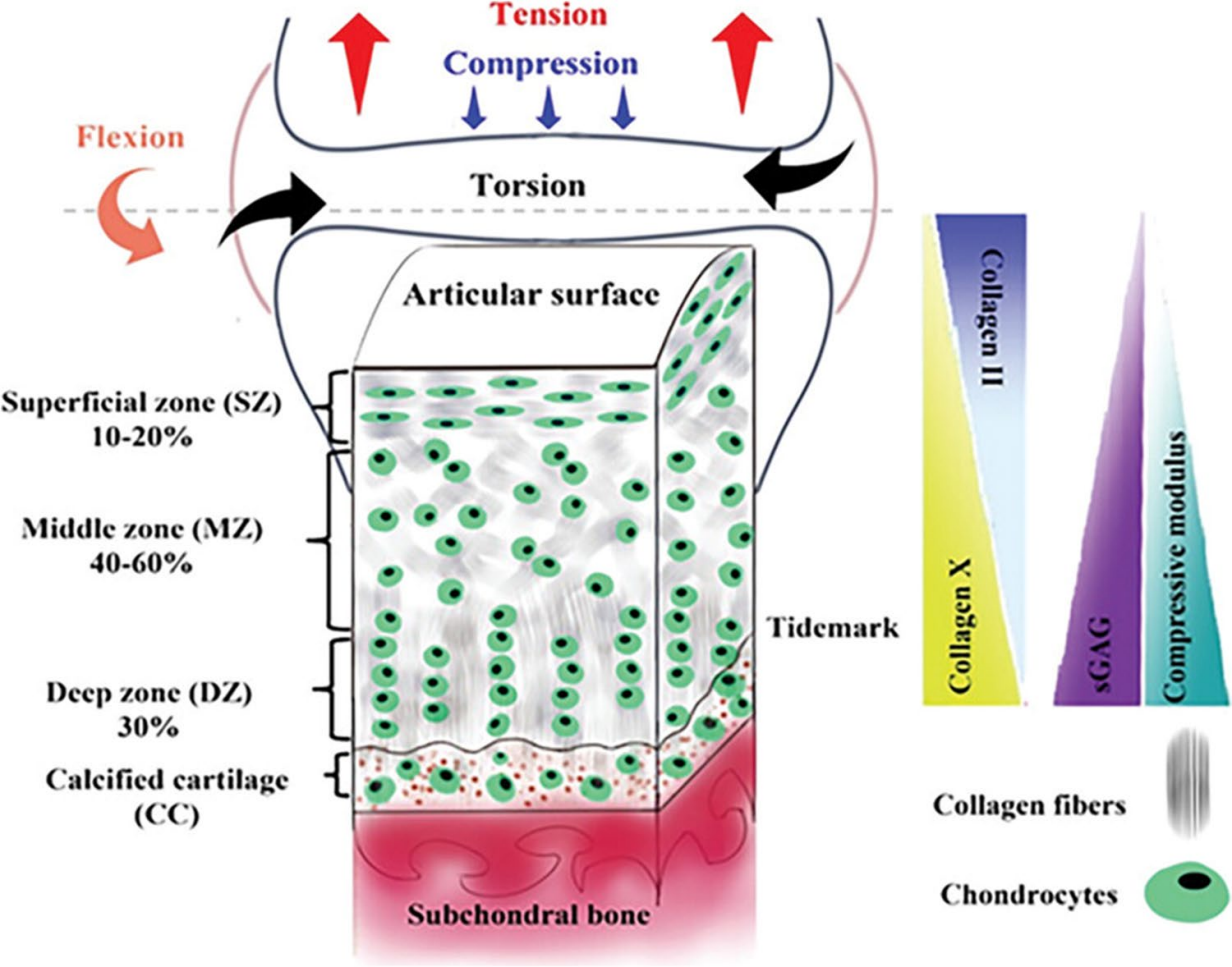
Structure and biochemistry of articular cartilage. Hyaline cartilage consists of a multi-layered structure with chondrocytes and a large extracellular matrix composed primarily of water with electrolytes, collagen fibrils, and highly negatively charged aggregates of proteoglycans (PGs). Collagen fibers (mainly type II) interact electrostatically with the glycosaminoglycans (GAGs) on PGs to form a cross-linked matrix. Biomechanically, the collagen network provides tensile stiffness to the tissue and the PG provides compressive stiffness. The collagen fibers are oriented parallel to the articular surface in the superficial zone, arcade-like in the transitional zone, and perpendicular in the radial zone. The water concentration differs slightly between zones ranging from 82% in the superficial zone to 76% in the radial zone. The PG concentration increases from the superficial to the deep zones. Figure revised from Dehghan-Baniani D. et al. Recent Advances in “Functional Engineering of Articular Cartilage Zones by Polymeric Biomaterials Mediated with Physical, Mechanical, and Biological/Chemical Cues.” *Adv Healthc Mater*. Apr 2023;12(10):e2202581 with permission

Articular cartilage plays a crucial role in sustaining joint health, mobility, and function. Supported by its unique, complex structure, cartilage facilitates load transmission while providing minimal friction for articulation (Fig. 1). Cartilage damage and degeneration are among hallmark manifestations of joint injuries and arthritis, classically osteoarthritis (OA). Cartilage imaging has transitioned from indirect imaging by radiographs to direct imaging by magnetic resonance imaging (MRI), and cartilage evaluation is now a standard component of all clinical joint MRI studies [1]. Cartilage compositional MRI (Cart-C MRI) refers to quantitative MRI measurements that reflect the biochemical components and organization of the cartilage microstructure such as proteoglycan (PG), water, and collagen content, e.g., the measurement of MR relaxation times. Comparatively, quantitative morphologic imaging of cartilage (Cart-M MRI) aims to measure articular cartilage thicknesses and volumes of focal or generalized regions of the articular cartilage. The significant advances in Cart-M MRI are beyond the focus of this review [2].

Cart-C MRI began in the 1990s, aiming to detect cartilage matrix changes from injury and degeneration in the earliest stages when there is relative preservation of tissue with minimal to no visible alterations on standard MRI. Subsequently, the methods were applied to postoperative assessment of surgical cartilage repair tissue. However, despite the significant advancements over the past three decades, Cart-C MRI remains predominantly a research tool, hindered by various technical and clinical hurdles. This review will commence with an overview of the technical evolution of Cart-C MRI, including advanced sequence development, accelerated acquisition techniques, and automated analysis using artificial intelligence (AI). We will then delve into the clinical applications of Cart-C MRI, including cohort studies, clinical trials, and its use at ultra-high field strengths. The review will conclude by identifying the existing gaps and challenges that need to be addressed to enable even broader clinical application of Cart-C MRI.

## Technical developments for Cart-C MRI

Articular hyaline cartilage consists of a low density of chondrocytes that are surrounded by a large amount of extracellular matrix (ECM) composed of water in a mixture of solid components that includes proteoglycans (PGs, predominantly aggrecans in cartilage), and collagen fibers (predominantly type II in hyaline cartilage). Degeneration of cartilage is characterized by progressive breakdown of the components of the cartilage ECM, resulting from an imbalance between anabolic and catabolic processes that are predominately controlled by the chondrocytes [3]. In the earliest phase of cartilage degeneration, the stage which Cart-C MRI techniques aim to detect, alterations of collagen structure and contents, loss of proteoglycan, and the consequent increase of water (tissue swelling) occur before there is cartilage thinning and tissue loss. Due to the very fast signal decay of protons in macromolecules, in vivo Cart-C MRI typically employs a strategy that “sensitizes” the measurement of relatively free water protons in the cartilage matrix to changes in macromolecular structure and content [4–6]. Depending on the principles for this sensitization, Cart-C MRI techniques can be categorized into the following groups: (1) relaxometry; (2) diffusion imaging; (3) magnetization transfer (MT) including chemical exchange saturation transfer (CEST) imaging, and lastly (4) non-proton, sodium MRI. Table 1 summarizes the basic principles, measurements, advantages, and limitations of the major Cart-C MRI techniques. In this section, we will focus on the non-contrast relaxometry techniques, which are closest to clinical translation, and discuss recent developments including advanced sequence development, accelerated acquisition, and automated analysis.

**Table 1:** Summary of major cartilage compositional MRI techniques

Cart-C MRI	Principles	Measurements	Advantages	Limitations
T_2_ [7, 8]	Spin–spin relaxation dominated by fibrous collagen network and water content	Single-echo spin-echo (gold reference) and variants for fast imaging (TSE/FSE, MESE), CPMG-prepared sequences, dual or triple echo steady state (DESS/TESS)	Sensitive to collagen structure and water content changes	Magic angle effect, less sensitive to proteoglycan, non-specific effects by water content and the collagen-PG matrix
T_1ρ_ [9–11]	Spin–lattice relaxation in the rotating frame with contributions from chemical exchange between protons of GAG and free bulk water, and from dipolar interactions	Spin-lock preparation followed by 2D or 3D readout	Sensitive to loss of proteoglycan. T_1ρ_ dispersion also provide tissue specific information	Relatively high SAR with spin-lock pulses, non-specific effects by water content and the collage-PG matrix, magic angle effects at low spin-lock frequency
T_2_*/UTE-T2* [12]	Composite of T_2_ decay and signal decay caused by local field inhomogeneity	3D spoiled gradient-echo, ultra-short TE (UTE)	Fast 3D imaging with high resolution; sensitive to cartilage matrix changes that will change local field inhomogeneity	Confounded by non-pathological factors that introduce local field inhomogeneity
dGEMRIC [13, 14]	Spin–lattice relaxation (T_1_) measurements in the presence of ionic MR contrast agent (dGEMRIC index) are proportional to the local glycosaminoglycan (GAG) concentration	T1 measured with inversion recovery (gold reference), look-locker, or variable flip angle methods	Sensitive and specific to fixed charge density (proteoglycan concentration)	Intravenous or intra-articular injection (usually double dose) of contrast agent with potential for side effects, waiting time required for contrast agent equilibration within the cartilage, potential incomplete and uneven contrast equilibrium in different cartilage regions
Diffusion [15, 16]	Anisotropic Brownian motion of water molecules in collagen-proteoglycan matrix is greater in damaged cartilage	Radial spin-echo diffusion tensor imaging, line-scan DTI (commonly available single short EPI DTI is not suitable for cartilage imaging)	Diffusivity inversely correlated with proteoglycan concentration; fractional anisotropy correlated with collagen structure	Low SNR in cartilage due to short T_2_, requirement for high resolution, sensitivity to motion
MT [17, 18]	Magnetization exchange between protons associated with semisolid macromolecules and free bulk water protons via dipolar coupling or chemical exchange	Signal differences in bulk water protons before and after saturation of macromolecule associated protons	Detect changes related to proteoglycan and collagen	Relatively high SAR with saturation pulses, non-specific effects by both proteoglycan and collagen
gagCEST [19]	Saturation transfer upon chemical exchange between protons associated with mobile compounds on GAG and free bulk water	Z-spectra with selective saturations of exchangeable proton groups on GAG side chains	Sensitive and specific to proteoglycan changes	Ultra-high field (≥ 7 T) best due to low CEST effect at lower field, susceptible to B_0_ inhomogeneity and other confounding factors including pH and cartilage T2
Sodium MRI [20, 21]	Distribution of positively charged sodium is proportional to the fixed charge density and local GAG concentration	Preferably 3D spoiled gradient-echo with radial k-space sampling for Ultra-short TE (TE < 0.1 ms)	Sensitive and specific to fixed charge density (proteoglycan concentration)	Low SNR, low spatial resolution, special hardware needed, prolonged scan time, best at ultra-high field (≥ 7 T)

*UTE* ultra-short time of echo, *dGEMRIC* delayed gadolinium-enhanced MR in cartilage, *MT* magnetization transfer, *CEST* chemical exchange saturation transfer. Techniques listed in the lower four rows are primarily research

Selected references cover some early work on ex vivo and in vivo technical development for each method

### Relaxometry without contrast—T_2_, T_1ρ_, and T_2_* mapping in cartilage

Relaxation times in the transverse plane, including T_2_, T_1ρ_, and T_2_*, are sensitive to activities of the water protons restricted within the collagen-PG matrix of cartilage and can be used to detect changes in cartilage collagen and PG [22]. Due to varying underlying mechanisms, each of these relaxation times has a different sensitivity to the various tissue constituents involved in cartilage degeneration.

*T*_*2*_, *the spin–spin relaxation*, of articular cartilage is dominated by extracellular cartilage water content and collagen structure. T_2_ values are modulated by the angle between collagen fibers and the magnetic field B_0_, which explains the magic angle effect of T_2_ (T_2_ is the longest when collagen fibers are oriented at the magic angle of 54.7°) [7].

Measurement of T_2_ requires collecting multiple spin echo images with different TEs, classically 2D multi-echo spin-echo (MESE) acquisitions. More recently, Carr-Purcell-Meiboom-Gill (CPMG) T_2_ prepared 3D gradient echo sequences have provided more efficient image collection. Although spin echo images are in general robust to B_0_ inhomogeneity, both methods can suffer from the error induced by imperfect refocusing pulse, resulting in stimulated echoes in MESE and T_1_ contamination in T_2_ preparation sequences. To overcome such B_1_-related error, extended phase graph (EPG) method is utilized to correct for the stimulated echo induced error in MESE [23], and more sophisticated refocusing pulse train such as Malcolm Levitt’s composite pulse decoupling sequence (MLEV) is used alongside with TE correction for T_2_-prepared sequences [24]. 3D double-echo steady-state (DESS) sequence can generate T_2_ mapping through either analytical approaches [25] or dictionary-based iterative procedures [26]. Simultaneous T_1_ and T_2_ estimates based on triple-echo steady-state (TESS) or multiple-echo steady-state (MESS) imaging have also been developed for efficient T_1_ and T_2_ measurements [27, 28]. Currently, the 2D MESE T2 mapping techniques are available as product sequences on clinical MR systems.

*T*_2_* mapping has also been applied in cartilage. T_2_* is sensitive to change in T_2_ as well as global and local inhomogeneity of the magnetic field. Alterations in T_2_* due to microscopic inhomogeneity can reflect tissue structural properties and provide information that is not present in T_2_ measures. However, higher sensitivity to susceptibility artifacts and imperfect magnet shimming present technical challenges for T_2_* imaging and complicate data interpretation. Unlike T_2_ imaging, T_2_* imaging uses gradient echoes with small flip angles (also available as product sequences on clinical MR systems) rather than spin echoes which permits fast acquisition. This allows for 3D acquisition and higher spatial resolution within a clinically relevant imaging time. However, T_2_* imaging for evaluating cartilage health is less established as both longer and shorter T2* in degenerated cartilage have been reported [29, 30]. Such discrepancies, caused by either different imaging protocols used or the real differences in matrix changes associated with the specific specimens/cohorts studied, present challenges when interpreting cartilage T_2_*. Combined with ultra-short TE (UTE) techniques, UTE-T2* imaging enables compositional evaluation of tissues with very short T_2_ and T_2_*, such as the deep cartilage layer, menisci, ligaments, and tendons [31, 32].

**Fig. 2:**
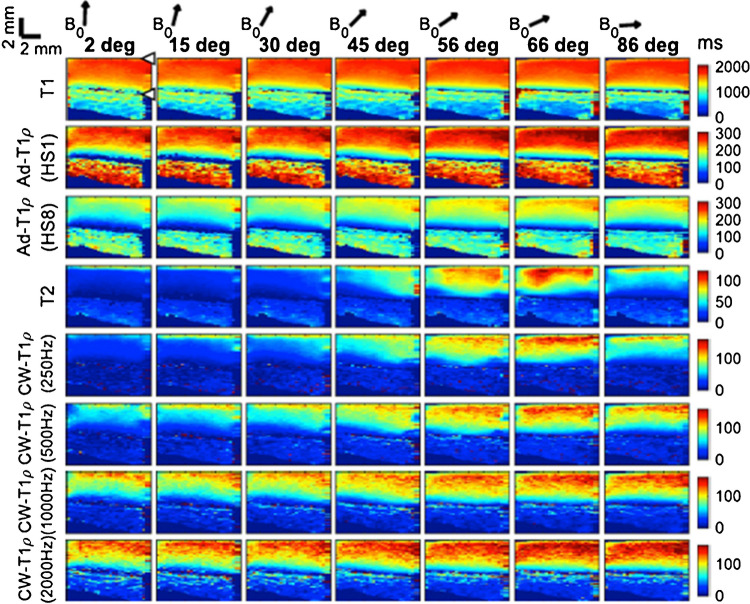
Relaxation parameter maps for a representative osteochondral sample imaged ex vivo at different angles with respect to B0 (arrows above). Orientation anisotropy is clearly seen for T_2_ and continuous wave (CW)-T_1ρ_ with low spin-lock strength (rows 4 and 5 from top). With increasing spin-lock strength (rows 6–8), there is progressively less anisotropy. Articular surface and cartilage-bone interface are marked with arrowheads. Adiabatic T_1ρ_ showed less anisotropy compared to CW-T_1ρ_. Figure revised from Reference 23 with permission

*T*_*1ρ*_, *the spin–lattice relaxation in the rotating frame*, is normally measured with spin-lock (SL) techniques. Chemical exchange between protons on the protein side chain groups of glycosaminoglycan (GAG) and free water has been attributed to T_1ρ_ relaxation and dominate the low-frequency (0–1.5 kHz) T_1ρ_ dispersion [33, 34]. T_1ρ_ dispersion is the phenomenon that T_1ρ_ increases with SL frequency; T_2_ can be considered T_1ρ_ at a spin-lock frequency of zero. T_1ρ_ has less dependence on collagen fiber orientation compared to T_2_ due to reduced dipolar interactions with SL techniques. In particular, T_1ρ_ collagen orientation dependency is minimized when the SL frequency is higher than 2k Hz (Fig. 2) [35]. For clinical imaging, the SL strength is normally limited to 500 Hz because of constraints on radiofrequency power deposition to the tissue, i.e., the allowable specific absorption rate (SAR), and hardware limitations. Therefore, clinical cartilage T_1ρ_ imaging is still subject to orientation dependency, although to a lesser extent than T_2_. T_1ρ_ measurements have shown superior to T_2_ measurements in differentiating between OA patients and healthy controls, especially for early and mild OA, potentially because of better sensitivity of T_1ρ_ to PG loss at early stages of the disease [36–39].

T_1ρ_ imaging sequences are composed of T_1ρ_ preparation with SL pulses, followed by gradient or spin echo readout. Continuous wave (CW) SL and adiabatic SL methods have been developed. CW SL locks the magnetization with a constant RF amplitude, which defines the SL frequency. Different pulse schemes have been employed to lessen B_0_ and B_1_ inhomogeneity-related errors including self-compensation phase cycling of spin-lock pulses, use of refocusing pulses, composite tip down/tip up pulses, and adiabatic tip down/tip up pulses [11, 40–44]. Kim et al. and Pala et al. recently compared robustness to B_0_ and B_1_ inhomogeneity of T_1ρ_ preparation schemes [45, 46]. Adiabatic SL pulses lock the magnetization through amplitude and frequency modulated RF pulses, and is less sensitive to B_0_ and B_1_ inhomogeneity artifacts [47]. However, the large RF energy deposition of adiabatic pulse is problematic, and the relaxation along fictitious field (RAFF) technique was developed to lessen the SAR burden [48].

T_1ρ_ imaging readout techniques began with 2D turbo spin echo (TSE) and spiral imaging; however, currently, 3D imaging techniques with multiple spoiled gradient echo acquisitions are most commonly used. Magnetization-prepared angle-modulated partitioned-k-space spoiled gradient-echo snapshots (termed MAPSS) T_1ρ_ imaging addresses several common issues during multiple gradient echo acquisitions [11, 49, 50]. RF cycling with two acquisitions per SL is applied to eliminate the T_1_ recovery contamination during the readout train. This RF cycling scheme also yields a transient signal evolution that is independent of the prepared magnetization. A variable flip angle train is then applied which provides a flat signal response and eliminates the filtering effect in k-space caused by transient signal evolution which improves quantification accuracy [11]. The MAPSS acquisition has been extended to T_2_ imaging with CPMG or MLEV T_2_ preparation, and allows simultaneous acquisition of T_1ρ_ and T_2_ mapping [49]. More recently, Peng et al. has demonstrated that a novel unpaired phase-cycling strategy with complex-value curve-fitting can eliminate signal contaminations from T_1_ recovery and allow for accurate quantitative parameter mapping with halved scan time compared to the original MAPSS strategy [51]. T_1ρ_ dispersion experiments with multiple SL frequencies (including R_2_-R_1ρ_, with R_2_ as R_1ρ_ at SL frequency = 0) may provide more specific information regarding chemical exchange and PG concentration [52]. Very recently, Han et al. proposed a novel method of using one pair T_1ρ_-weighted and T_2_-weighted images to estimate R_2_-R_1ρ_, with an optimal T_prep_ considering cartilage relaxation times and image signal-to-noise ratio (SNR) [53]. Currently, T_1ρ_ mapping sequences are only available as research prototypes on clinical MR systems.

### Accelerated Cart-C MRI acquisition

Acquisition acceleration has been a crucial development in Cart-C MRI, given the typically lengthy imaging times required for obtaining multiple images for quantifying compositional parameters.

*Parallel imaging* techniques take advantage of multi-channel coils; however, the acceleration factor (AF) is primarily limited by the number and geometry of coil channels and resulted reduced SNR in reconstructed images (note: AF indicates the scan time reduction. For example, AF = 2 reduces scan time approximately half, while AF = 3 reduces scan time approximately to 1/3 of the original time). Pakin et al. and Zuo et al. showed that a maximum AF = 3 can be achieved with parallel imaging for cartilage T_1ρ_ and T_2_ mapping without sacrificing quantitative accuracy [54, 55].

**Fig. 3:**
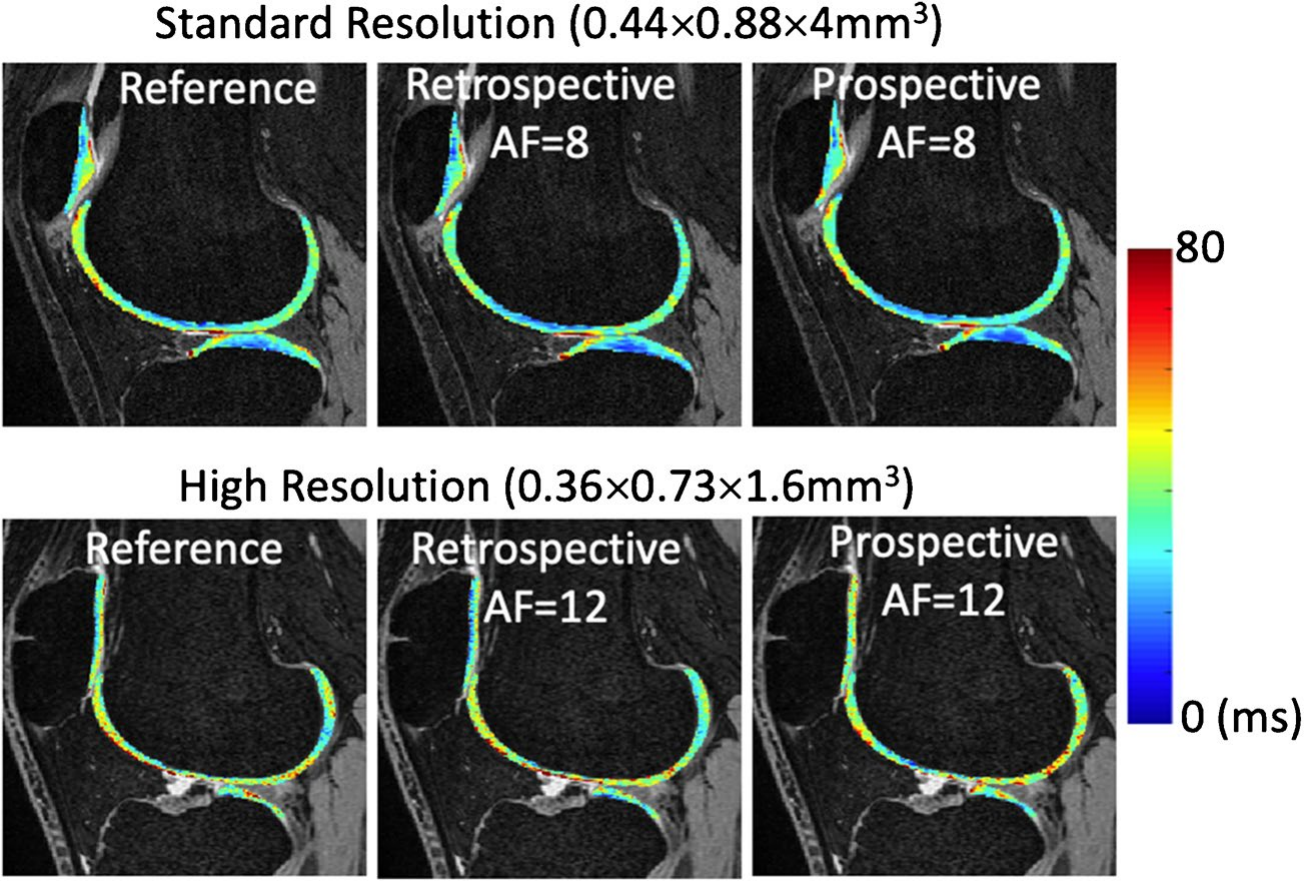
Cartilage T_1ρ_ maps with accelerated imaging using compressed sensing reconstruction demonstrates excellent agreement with reference maps for both retrospective and prospective downsampling. For standard resolution imaging (top row), the average coefficient of variation (CV) between reference and AF8 maps was 2.36% and 3.07% for retrospective and prospective downsampling, respectively (*n* = 5). For high-resolution imaging (bottom row), the average CV between reference and AF12 maps was 2.32% and 1.05% for retrospective and prospective downsampling, respectively (*n* = 3). AF, acceleration factor

*Compressed sensing* (CS) techniques take advantage of image sparsity and use incoherent k-space data undersampling, which allows higher AFs [56]. Huang et al. proposed to combine principal component analysis (PCA) across the temporal dimension with a model-based algorithm to reconstruct T_2_ maps [57], while Peng et al. exploited the linear predictability of the T_2_ exponential delay on top of the low-rank and joint-sparsity constraints [58]. Both studies reported good agreement with reference maps using AF = 8 for knee cartilage. Zhou et al. used iteratively local support detect (k-t LAISD) to improve CS reconstruction in cartilage T_1ρ_ imaging [59]. Zhu et al. applied PCA as a sparsifying transform and the T_1ρ_ images were reconstructed using dictionary learning [60]. Pandit et al. combined CS with parallel imaging, where the data collected from multiple coils were used [61]. Zibetti et al. compared 12 different sparsifying transforms in CS to accelerate 3D-T_1ρ_ imaging and suggested that spatial–temporal finite-difference (STFD) regularization had the best results [62]. Using STFD CS reconstruction, Kim et al. achieved AF = 8 for simultaneous T_1ρ_ and T_2_ imaging with standard resolution (0.44 × 0.88 × 4mm^3^), and AF = 12 for high-resolution T_1ρ_ imaging (0.36 × 0.73 × 1.6mm^3^) in a multi-vendor multi-site study, with good agreement to reference maps for both retrospective and prospective undersampling (Fig. 3) [63].

**Fig. 4:**
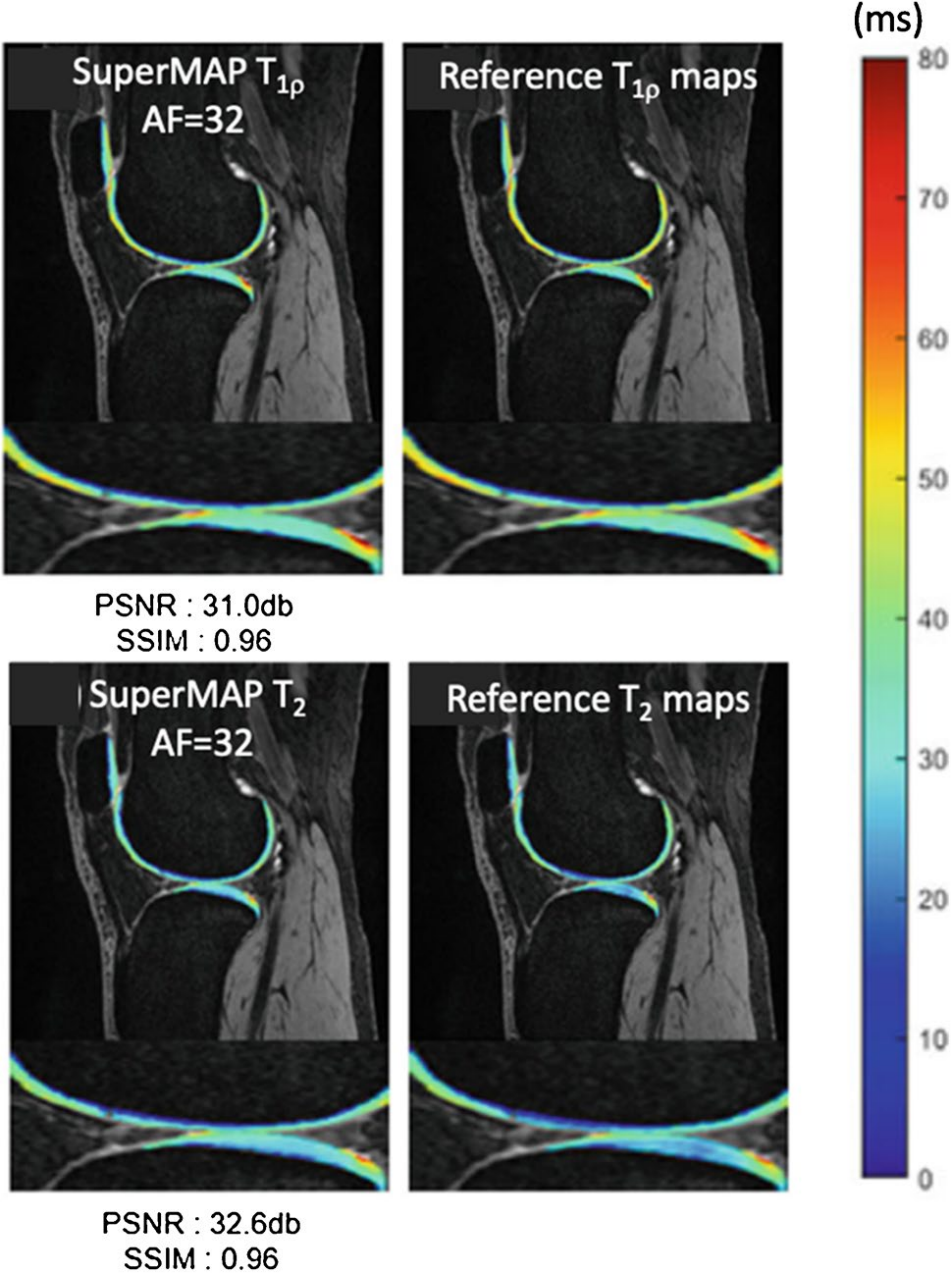
Cartilage T_1ρ_ (top) and T_2_ (bottom) maps reconstructed using deep learning-based reconstruction with joint spatiotemporal undersampling (superMAP) show excellent agreement with reference maps acquired with the MAPSS sequence. With this technique, it is possible to acquire 3D T_1ρ_ and T_2_ maps of the whole knee within 2 min. AF, acceleration factor. PSNR, peak signal to noise ratio. SSIM, structural similarity index measure. Figure revised from Reference 59 with permission

*Deep learning-based reconstruction* can accelerate cartilage relaxometry mapping acquisition. For example, variational networks have been used to reconstruct T_1ρ_ echo images from subsampled k-space data [64]. The subsequently cartilage T_1ρ_ maps generated by monoexponential and biexponential fitting produced more accurate T_1ρ_ maps compared to using CS [64]. Tolpadi et al. developed a recurrent U-Net pipeline with region-of-interest-specific loss functions to yield robust T_2_ maps of small, clinically crucial features such as cartilage of knee and hip, and intervertebral discs, from accelerated MAPSS acquisition with AF up to 12 [65]. Very recently, Li et al. demonstrated the feasibility of utilizing deep learning to accelerate T_1ρ_ and T_2_ mapping acquisition via joint spatiotemporal undersampling (termed as superMAP). Acceleration factors as high as 32 were obtained for retrospective undersampling and AF = 26 for prospective undersampling to simultaneously reconstruct T_1ρ_ and T_2_ maps from MAPSS combined T_1ρ_ and T_2_ acquisition (Fig. 4) [66]. With this technique, it would be possible to acquire accurate 3D T_1ρ_ and T_2_ maps of the whole knee within 2 min, a clinically feasible acquisition time.

*MR Fingerprinting (MRF)* allows fast, simultaneous measurement of tissue properties including relaxation times for many anatomic locations [67]. However, the high spatial resolution required for MSK applications is a challenge for the technique, and sequence optimizations led to promising applications of MRF in cartilage T_1_, T_2_, and T_1ρ_ imaging [68–70]. The feasibility to further accelerate MRF acquisition for knee T_1_ and T_2_ mapping using deep learning reconstruction has been recently demonstrated [71]. Compared to conventional MRF, the authors were able to reduce the number of time frames from 1000 to 50 in addition to the AF of 15 in the k-space, without sacrificing the T_1_ and T_2_ map quality.

Currently, parallel imaging has been used as the default acceleration method for sequences including Cart-C MRI on clinical MR systems for data collected with multi-channel coils. Cart-C MRI product sequences with advanced accelerated techniques, such as compressed sensing and deep learning reconstruction, are primarily limited to research prototypes, except for 2D MESE T_2_ mapping. More efforts on implementing fast 3D Cart-C MRI mapping (< 5 min) with advanced reconstruction techniques as product sequences on clinical MR systems are warranted to integrate these advanced techniques into clinical protocols and facilitate their clinical translation.

### Automated analysis of Cart-C MRI

**Fig. 5:**
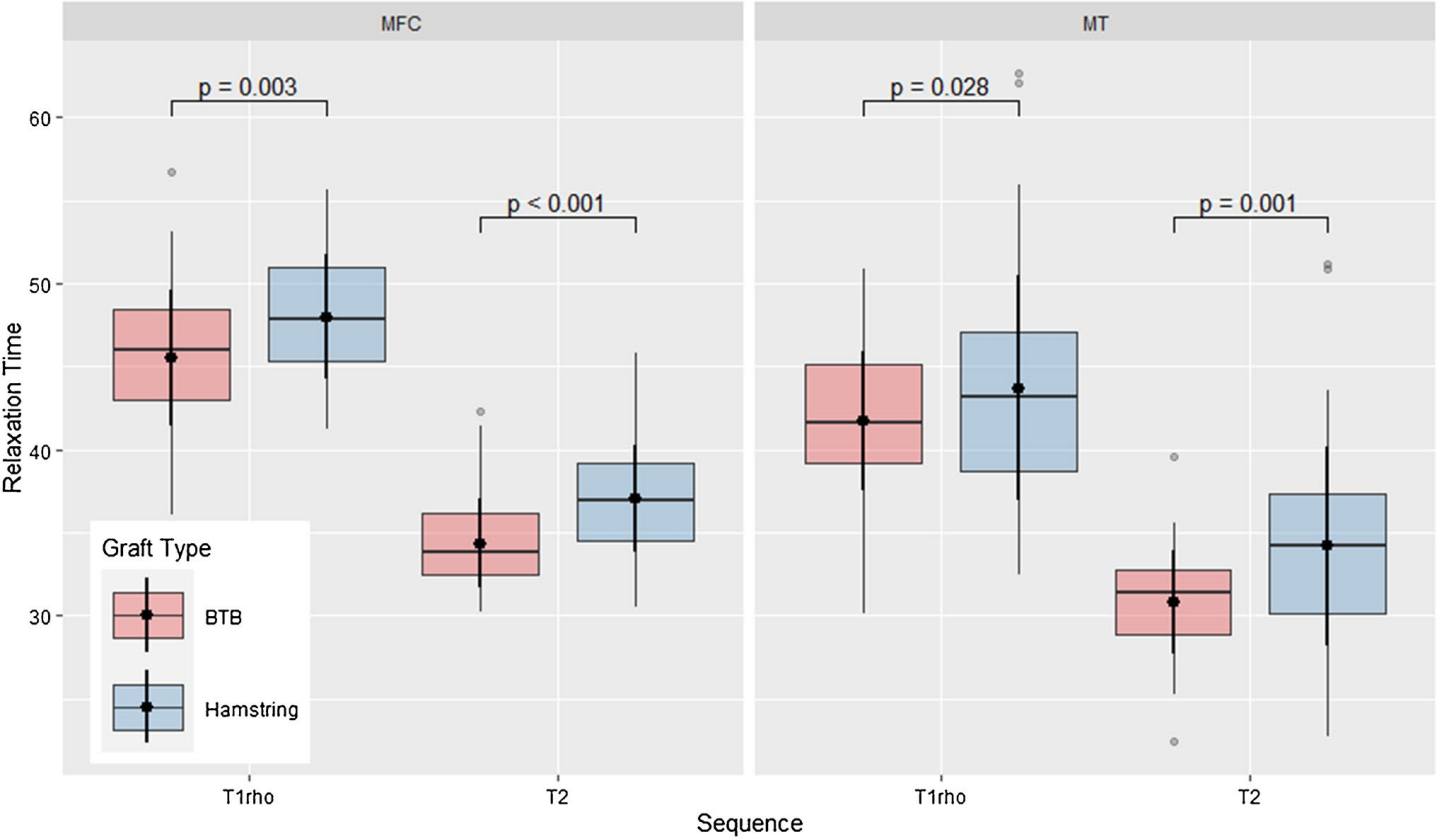
Comparison of cartilage T_1ρ_ and T_2_ values for the medial femoral condyle (MFC, left) and medial trochlea (MT, right) for patients > 10 years following ACL reconstruction using either hamstring autograft (*n* = 51) or bone-patella tendon-bone (BTB) autograft (*n* = 63). The hamstring autograft group showed significantly higher T_1ρ_ and T_2_ in both compartments compared to the BTB autograft, suggestive of greater cartilage degeneration for this group

Automated cartilage segmentation is the first step in the automated analysis of Cart-C MRI. Cartilage segmentation has evolved from manual segmentation, semi-automatic, to fully automatic methods, recently enabled by the deep learning-based methods [72, 73]. For instance, Gaj et al. developed a U-Net-based generative adversarial networks (GAN) for automated and accurate cartilage segmentation on DESS images from the Osteoarthritis Initiative (OAI) dataset [74]. Very briefly, a GAN trains two neural networks to compete against each other to generate more authentic new data from a given training dataset. Holden et al. adopted this model to automatically segment cartilage to study potential baseline predictors for cartilage T_1ρ_ and T_2_ at 10 years after anterior cruciate ligament reconstruction (ACLR), where they observed that hamstring autograft had greater cartilage T_1ρ_ and T_2_ values compared to bone-patella tendon-bone autograft (Fig. 5) [75]. Xue et al. used a modified U-Net to automate cartilage segmentation for relaxometry quantification using 3D UTE-cones sequences and showed the method differentiated healthy control subjects from moderate to severe OA patients [76].

**Fig. 6:**
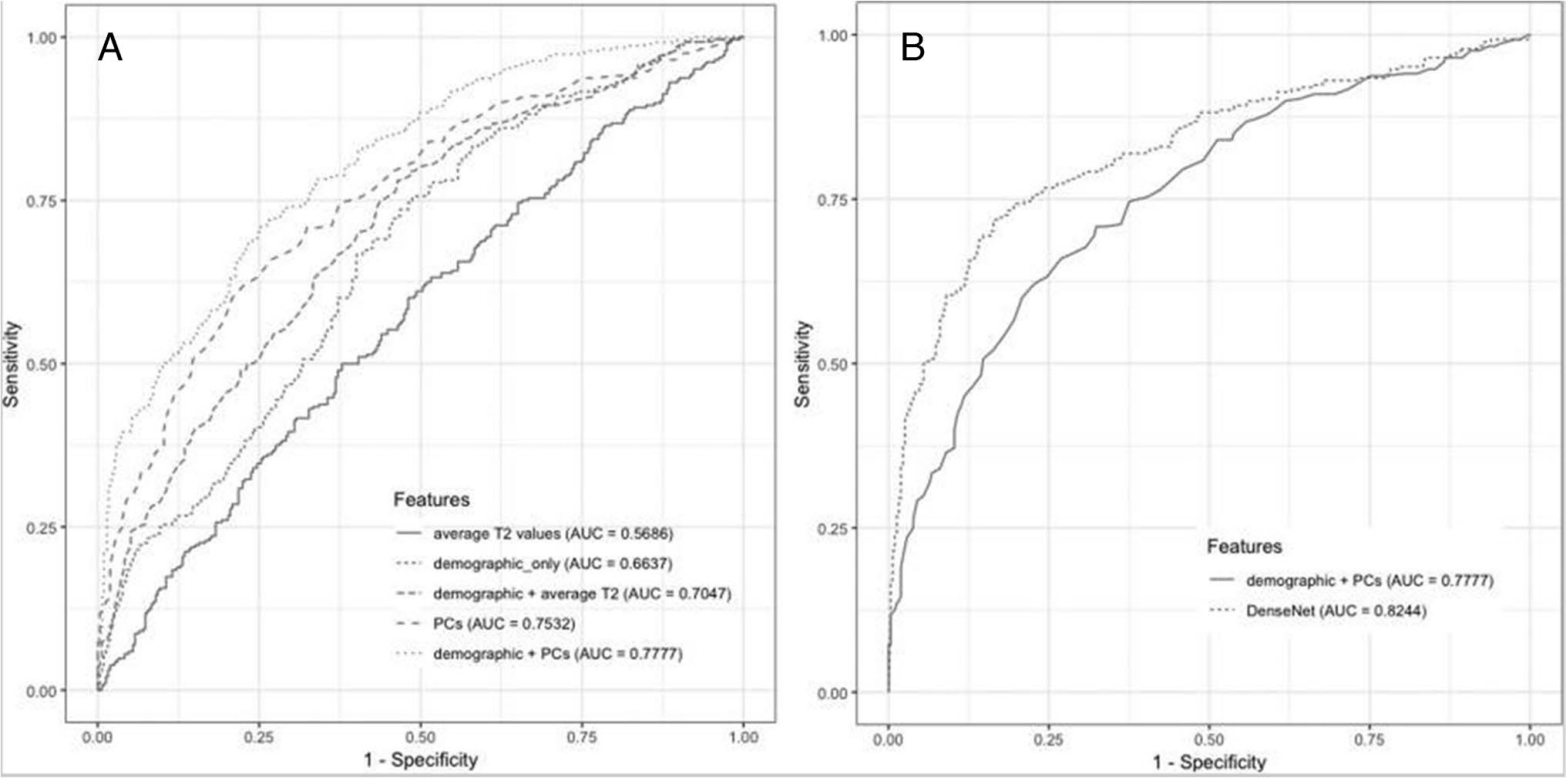
Diagnosing OA from T_2_ maps using the entire OAI baseline cohort (*n* = 4384). After a deep learning model was used for cartilage segmentation, a DenseNet directly applied to T_2_ maps was used to diagnose OA and compared to the diagnosis obtained using random forest applied on demographic information and dominant principal components from voxel based relaxometry analysis. **A** ROC curves comparing the random forest results between different feature combination. **B** ROC curves comparing the best performant shallow classifier with the deep learning model using DenseNet. DenseNet-based approach outperformed the conventional random forest approach with AUC = 0.8344. ROC, receiver operator characteristic; AUC, area under curve; PC, principal component. Figure from Reference 70 with permission

Pedoia et al. recently performed T_2_ analysis on the complete OAI baseline dataset using AI [77]. After a deep learning model was used for cartilage segmentation, a DenseNet directly applied to T_2_ maps was used to diagnose OA and compared to the diagnosis obtained using random forest applied on demographic information and dominant principal components from voxel based relaxometry analysis. They showed that the DenseNet-based approach outperformed the conventional random forest approach in terms of ROC AUC (Fig. 6).

**Fig. 7:**
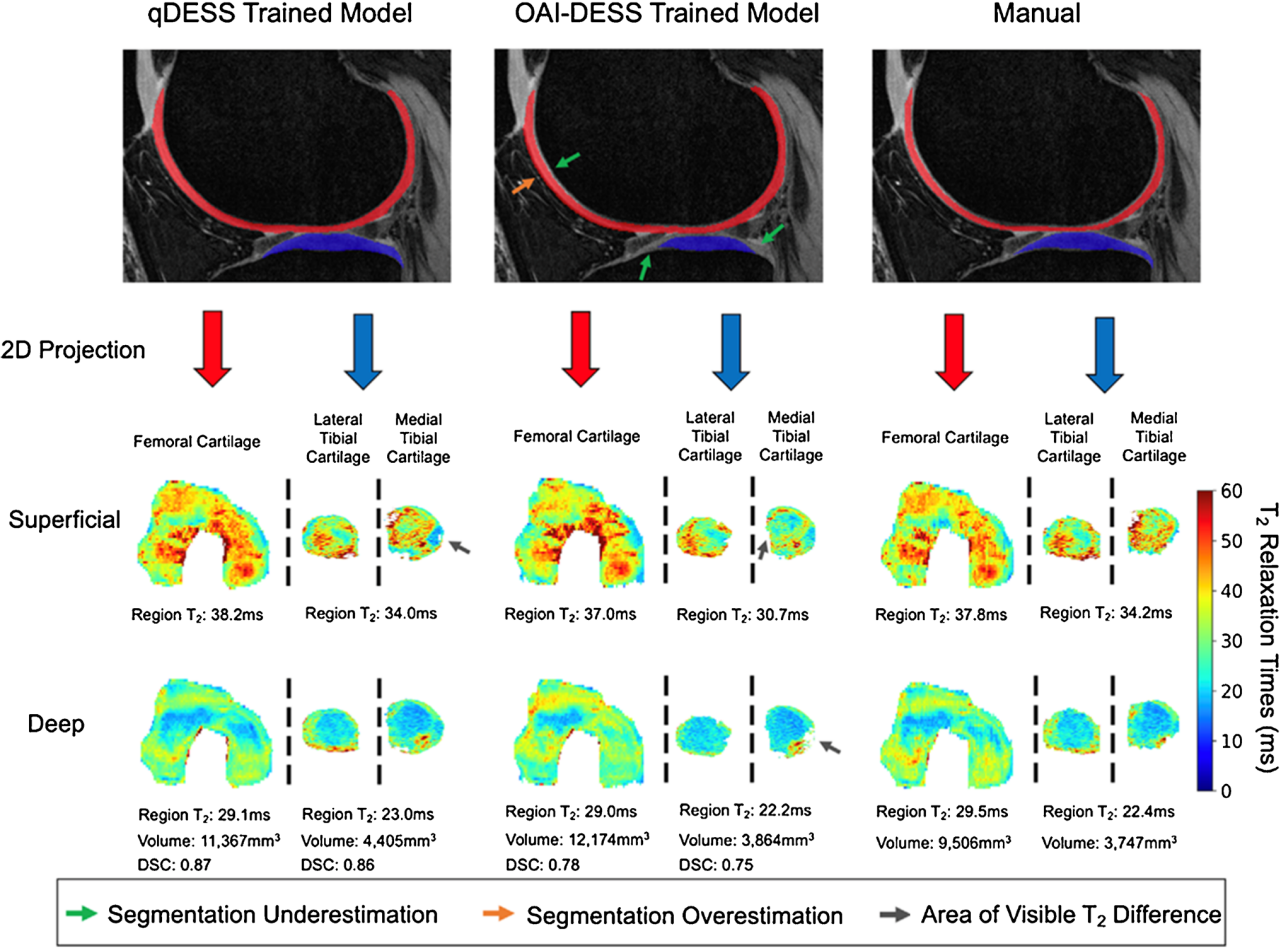
Comparison of manual and automatic segmentations from OAI- and qDESS-trained models and respective 2D unrolled T_2_ maps in the left knee of a subject. The subject’s T_2_ values from the superficial and deep cartilage regions, cartilage volumes, and DSC values for the qDESS- and OAI-DESS-trained models are also shown. Arrows indicate examples of visually apparent differences in the automated segmentations and resultant T_2_ maps. These differences typically appear at the periphery of cartilage surfaces, which have limited impact on subregion estimates. Figure from Reference 71 with permission

Schmidt et al. recently studied the generalizability of deep learning segmentation models, which can be problematic for models trained on limited image datasets [78]. The authors showed that a qDESS-trained model performed better than an OAI-trained model on the independent qDESS images from four study cohorts (a total of 59 subjects and 82 knees) with various KL grades (Fig. 7), which confirmed that the domain shift problem should be considered when applying deep learning models to different cohorts.

## Clinical applications of Cart-C MRI

Numerous ex vivo studies have demonstrated Cart-C MRI measures are correlated with biochemical, biomechanical, and histological analysis of cartilage specimens [79]. A systematic review and meta-analysis by Emanuel et al. showed that dGEMRIC and T_1ρ_ are significantly correlated to PG concentrations, while T_2_ had a weaker correlation with PG and showed the relationship with collagen was greater for fiber organization than collagen concentration [80]. These validation studies are the foundation for proposing Cart-C MRI techniques as potential imaging biomarkers for disease. In human studies, Cart-C MRI techniques have shown good to excellent scan-rescan reproducibility. A systematic review and meta-analysis by Mackay et al. reported that for T_2_, T_1ρ_, and dGEMRIC, most test/retest intraclass correlation coefficients were greater than 0.8 and coefficients of variation less than 10% [39]. Based on its ex vivo validity and in vivo reproducibility, Cart-C MRI has become not only a research tool for exploring cartilage physiology, microstructure, and degeneration process, but, more importantly, a powerful instrument to provide clinically relevant evaluations [39, 81]. In this section, we will discuss the applications of Cart-C MRI, including cohort studies, clinical trials, patient treatment planning, and finally, Cart-C MRI at ultra-high field. The discussion is focused on the knee, but Cart-C MRI has also been applied in other joints including the hip, ankle, hand/wrist, shoulder, and intervertebral discs [22, 82].

### Natural history observational cohort—the Osteoarthritis Initiative (OAI)

**Fig. 8:**
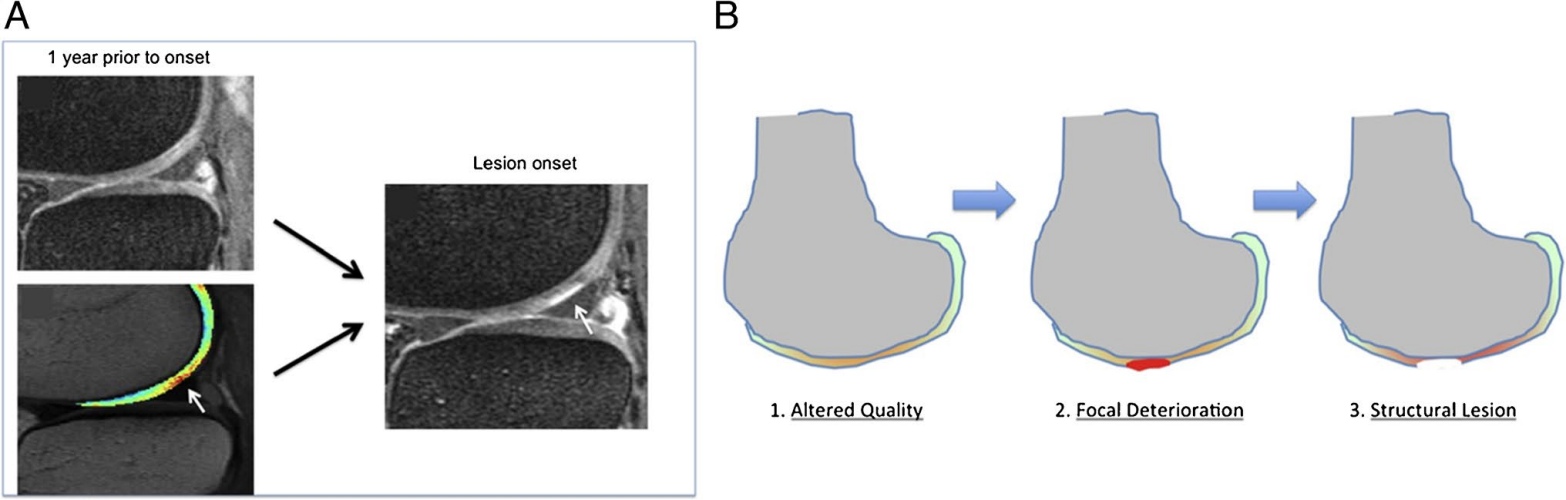
Spatial–temporal relationship between cartilage T2 elevation and cartilage focal lesion development. **A** An example patient who demonstrated cartilage lesion development. While the cartilage of the lateral femoral cartilage appears normal in the sagittal fat-saturated intermediate weighted sequence (left top), local T2 elevation of the cartilage T2 map (left bottom, white arrow) is demonstrated in the lesion equivalent area 12 months prior to lesion onset (right, white arrow). **B** Graphic illustration of compositional cartilage degradation prior to the onset of a macroscopic lesion proposed by the authors. Authors investigated cartilage plates from knees which developed new-onset cartilage lesions (*n* = 57) over a 4-year period, comparing against cartilage plates from control knees (*n* = 52) at both the focal lesion and cartilage plate level. The authors showed that, at the local level, cartilage T_2_ values were significantly higher in case knees at 1 year prior to lesion onset, and at 2 years prior to onset at the plate level. Figure revised from Reference 81 with permission

Cart-C MRI studies, often limited to small cohorts due to technical complexities and resource constraints, have been significantly expanded by the inclusion of T_2_ mapping in the OAI protocol. Initiated in 2004, the OAI is a multicenter, prospective, observational cohort study of knee OA that collected 8 years of longitudinal imaging, including T_2_ mapping, and clinical data from 4796 subjects at four sites [83]. Analyses of the OAI cohort data have identified T_2_ relaxation time as a marker for early-stage cartilage degeneration, as well as monitoring and predicting longitudinal disease progression [84, 85]. Joseph et al. showed that higher baseline cartilage T_2_ values and T_2_ heterogeneity were predictive of morphological degeneration of cartilage and meniscus, and bone marrow lesions over 3 years [86]. Kretzschmar et al. reported that areas that developed cartilage defects over a 4-year period demonstrated elevated cartilage T_2_ in the same location before the cartilage defects developed (Fig. 8) [87]. Using automated cartilage segmentation with AI, Razmjoo et al. analyzed cartilage T_2_ values for all OAI subjects (25,729 knee MRIs) and revealed that higher tibiofemoral T_2_ values significantly increased the likelihood of developing radiographic OA and the risk of having a total knee arthroplasty [88]. In addition, cartilage T_2_ values have been correlated with physical activity and weight loss both cross-sectionally and longitudinally in OAI subjects [89–91], suggesting that cartilage T_2_ can be used to monitor outcomes for these important interventions for OA management.

### Applications in sports medicine—imaging joint injuries and post-traumatic osteoarthritis

**Fig. 9:**
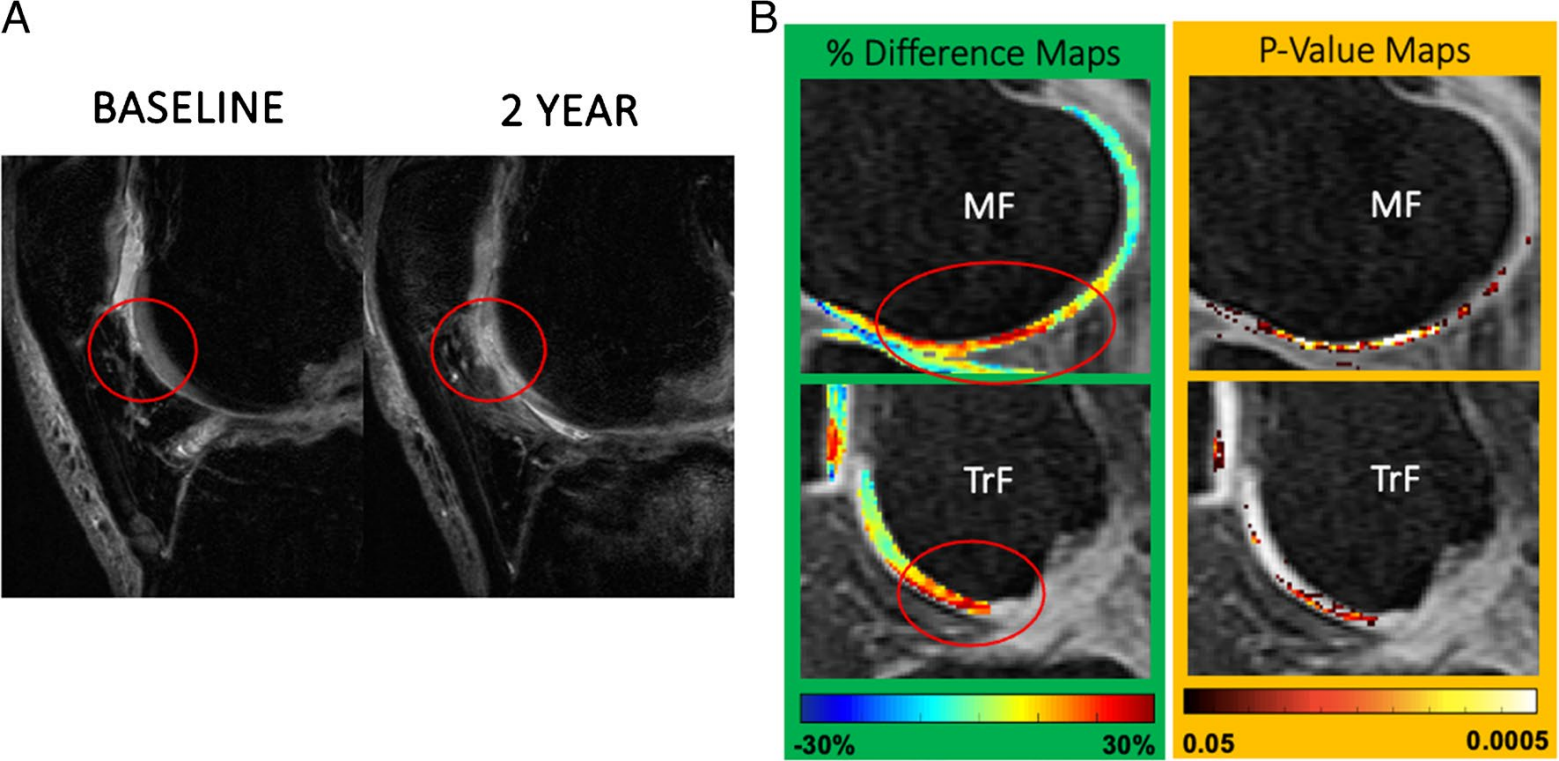
Voxel-based relaxometry (VBR) analysis demonstrated significant higher baseline (after ACL tear and before ACLR) cartilage T_1ρ_ values predicted cartilage lesion progression at 2 years after ACLR. **A** An example patient who showed patellofemoral joint (PFJ) cartilage lesion development at 2 years after ACLR (red circle). **B** Percent difference and *P*-value maps between the PFJ progression and non-progression groups. Differences were calculated as (progression–non‐progression). Significantly elevated T_1ρ_ values were observed in medial femoral (MF) and trochlea (TrF) cartilage (red circles) in the progression group. Figure revised from Reference 96 with permission

Acute joint injuries such as ACL tear, meniscal tear, patellar dislocation, intra-articular fracture, and ankle injury have shown as risk factors for post-traumatic osteoarthritis (PTOA) [92, 93]. Cart-C MRI provides a powerful tool for identifying the cartilage at risk for degeneration. Early identification of “cartilage at risk” with Cart-C MRI is a promising tool for the development and application of early interventions to prevent patients from progressing to permanent cartilage damage and subsequent PTOA. Cart-C MRI has been extensively studied in patients after ACL injury with a recent systematic review suggesting that T_1ρ_ and T_2_ are promising biomarkers for diagnosis and prediction of PTOA after ACLR [94]. Significantly elevated cartilage T_1ρ_, T_2_, and UTE-T_2_* and decreased dGEMRIC index have been observed after acute ACL injury and after ACLR [95–99], compared to contralateral or control knees. Pietrosimone et al. and Williams et al. reported significant correlation between cartilage T_1ρ_ and UTE-T_2_* values and patient outcomes as evaluated with KOOS at 1 year and 2 years after ACLR, respectively [100, 101]. Using voxel-based relaxometry (VBR) analysis, Xie et al. reported that baseline T_1ρ_ and T_2_ predicted cartilage lesion development 2 years after ACLR (Fig. 9) [102]. Baseline cartilage T_1ρ_ and T_2_ values have predicted patient outcomes evaluated by KOOS at 6, 12, and 24 months after ACLR [102, 103]. A lower dGEMRIC index of femoral cartilage measured 2 years following ACL rupture was found prognostic of both radiographic and symptomatic knee OA at 14 years [104].

Using T_1ρ_ and T_2_ as markers for the evaluation of cartilage health, researchers have identified correlations between cartilage health and factors that may contribute to PTOA development after ACL injury, including meniscal injury [105], bone shape [106], biomechanics after ACLR [107–109], physical activities [110], synovial fluid inflammatory biomarkers [111], and surgical factors such as time from injury to surgery, surgical technique, concomitant meniscal treatment [112, 113], and quadriceps femoris strength at the time of return to sports [114]. These studies suggested Cart-C MRI measures can serve as sensitive outcome measures for optimizing patient management after acute joint injuries and identifying risk factors for PTOA.

### Clinical applications following cartilage repair and regeneration

Another significant application of Cart-C MRI is postoperative assessment of all types of cartilage repair surgery [115], but particularly for the determination of the structure of the generated repair tissue that grows following procedures such as microfracture, autologous chondrocyte implantation (ACI), matrix-induced ACI (MACI), and particulated cartilage allografts [116, 117]. MRI is an ideal noninvasive modality for determining both morphological and compositional surgical outcomes. Two systemic reviews found significant correlation between Cart-C MRI, especially T_2_ mapping, and clinical outcomes following cartilage repair surgery, suggesting Cart-C MRI may offer a noninvasive method to monitor cartilage repair tissue that is clinically meaningful [118, 119].

**Fig. 10:**
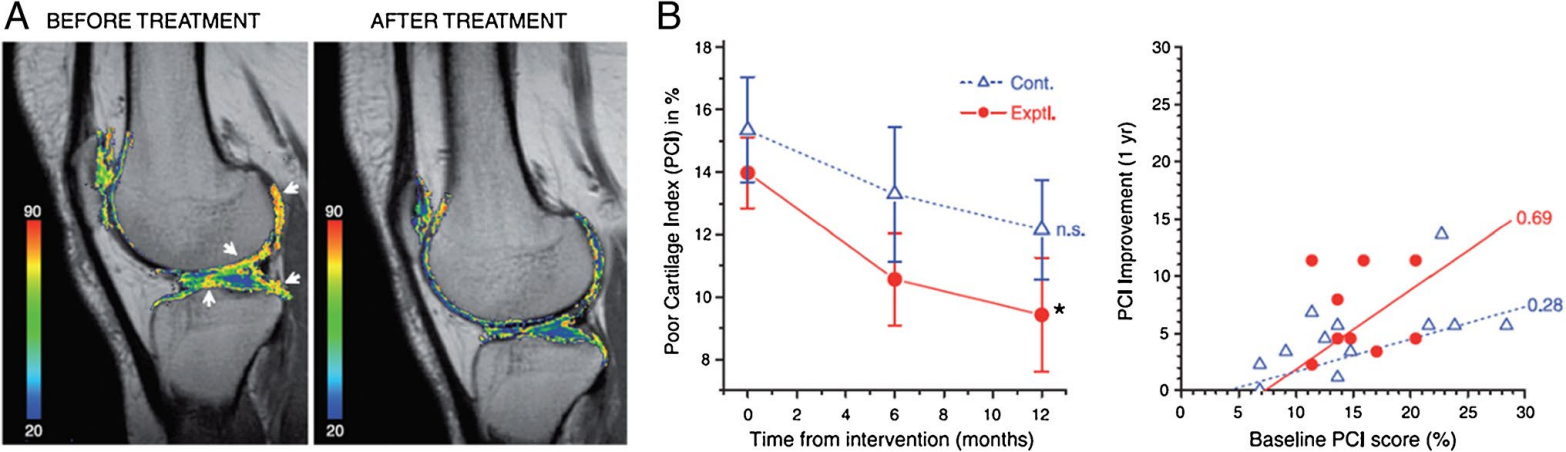
Monitoring articular cartilage quality with T_2_ mapping. **A** T_2_ maps of a patient before and after mesenchymal stem cell (MSC) treatment. Cartilage T_2_ values reduced in the indicated areas (white arrows) following MSC-treatment implying improvement in cartilage quality. **B** Cartilage quality, assessed by T_2_ mapping was quantified using the poor cartilage index (PCI, computed as the percentage of sample points with a T_2_ relaxation value > 50 ms). The worst possible value for PCI is 100, whereas healthy cartilage will approach 5. Left: temporal evolution of PCI, mean ± SE values of 12 patients treated with MSCs (filled circles; continuous line) and 15 active controls treated with hyaluronic acid (open triangles; dotted line), *P < 0.05 (repeated measures ANOVA with a Dunnett multiple test compared to the baseline), n.s. = nonsignificant. Right: The correlation between PCI improvement and the initial PCI score is shown for all the patients included in this study. Figure revised from Reference 113 with permission

Compositional MRI methods have been applied to evaluate cartilage regeneration following various interventions in randomized clinical trials. Vega et al. observed a significant reduction of cartilage T_2_ values (indicating improved cartilage health) following intra-articular injection of autologous mesenchymal stromal cells (MSCs) (30 patients, Kellgren Lawrence [KL] = 2–4) (Fig. 10) [120]. However, Chahal et al. reported no significant change in T_2_ values after autologous MSC treatment, despite the improvement in symptoms evaluated with KOOS and WOMAC (12 patients, KL = 3–4) [121].

Park et al. investigated the efficacy of allogeneic human umbilical cord blood-derived MSCs for cartilage regeneration in seven OA patients (KL = 3). The dGEMRIC analysis showed high GAG content in regenerated cartilage after 3 years, aligning with histological findings of the cartilage at 1 year [122]. Additionally, McAlindon et al. used dGEMRIC imaging to demonstrate short-term (6 months) changes in knee hyaline cartilage following collagen hydrolysate treatment in 30 patients with mild to moderate OA [123].

In a recent phase I/IIa randomized clinical trial, Zhao et al. applied a multi-compositional MRI approach to detect changes in cartilage composition after treatment with allogeneic human adipose-derived mesenchymal progenitor cells (haMPCs) (18 patients, KL = 2–3) [124]. Significant differences were observed in quantitative T_1ρ_, T_2_, T_2_*, and apparent diffusion coefficients (ADC) measurements between three dose groups, with T_1ρ_ being the most sensitive technique. Treated subjects showed significant improvements in WOMAC and SF-36 scores, suggesting the cartilage compositional changes may correlate with patient symptom alleviation [124].

### Cart-C MRI at ultra-high field

The approval of 7 Tesla (7T) MR systems for clinical use by the European Medicines Agency (EMA) and U.S. Food and Drug Administration (FDA) in 2017 has significantly enhanced the clinical utility of 7T MRI. 7T offers an increase in SNR, contrast-to-noise ratio (CNR), susceptibility effects, and spectral resolution. However, ultra-high field MRI also encounters challenges such as SAR limitations and non-uniform B_1_ transmit fields. Robust shimming and RF calibration methods are even more critical for accurate and reliable MR parameter quantification. Despite these issues, Cart-C MRI at 7T continues to evolve, with expanding applications as technical issues are resolved [125].

In *T*_*2*_* and T*_*1ρ*_* mapping*, the increased spatial resolution possible at 7T helps to alleviate partial volume averaging effects on quantitative results and improves the evaluation of thin cartilage, such as in the ankle and wrist as well as zonal comparison of deep and superficial cartilage T_2_. Domayer et al. found differences in zonal MESE T_2_ assessment between the cartilage of healthy volunteers and the cartilage repair tissue of patients after two different repair techniques in the ankle joint [126]. Another study reported that MESE T_2_ mapping of knee cartilage can not only distinguish between the healthy cartilage and cartilage repair tissue, but also between the healthy cartilage and the cartilage adjacent to the repair tissue with otherwise normal thickness [127]. Compared to MESE T_2_ mapping, T_2_ mapping techniques based on gradient echo acquisition, such as MAPSS and TESS, have mitigated the issue of SAR limitation and are less sensitive to B_1_ inhomogeneity at 7T [128, 129]. Lower cartilage T_2_ and T_1ρ_ were reported at 7T compared to 3T [130, 131], as expected (T_1_ increases with field strength and it is general accepted that T_2_ and T_1ρ_ decrease with field strength but at a smaller percentage compared to T_1_ increase); conversely, some investigations did not observe significant differences in cartilage between the two field strengths [128]. Wyatt et al. reported an average 60% higher SNR at 7T versus 3T and found larger differences in cartilage T_2_ and T_1ρ_ values between healthy subjects and OA patients at 7T than at 3T, suggesting greater sensitivity of T_2_ and T_1ρ_ mapping to cartilage degeneration at 7T compared to 3T at the same resolution [128].

**Fig. 11:**
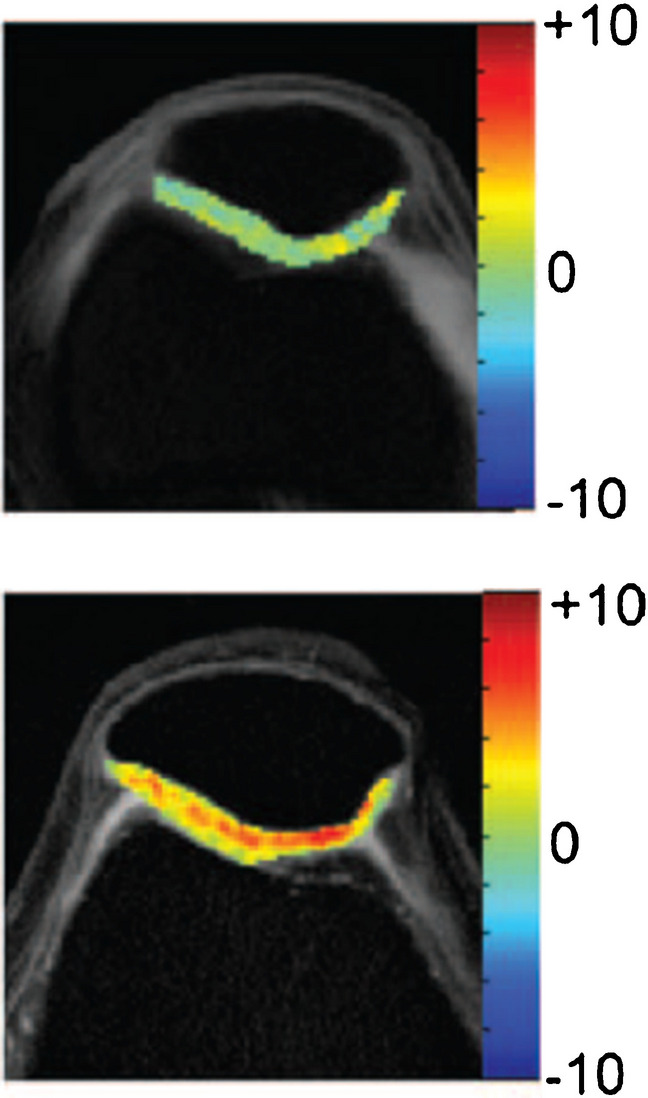
Cartilage gagCEST maps in a healthy volunteer acquired at 3 T (top) and at 7 T (bottom). The gagCEST effect is much higher at 7 T compared to 3 T. Color bar shows gagCEST asymmetry values. Figure revised from Reference 127 with permission

*Glycosaminoglycan chemical exchange saturation transfer* (gagCEST) greatly benefits from the improved selectivity of saturation RF pulses (because of greater frequency dispersion) at 7T as well as the improved SNR compared to 3T (Fig. 11) [132]. gagCEST relies on sophisticated post-processing including motion-, *B*_0_-, and *B*_1_-corrections [132]. Brinkhof et al. reported decreased gagCEST values in cartilage defects and good reproducibility when averaged over larger cartilage compartments [133]. However, gagCEST maps typically show a relatively large range of values even in healthy cartilage [133] that may limit clinical utility. Furthermore, cartilage T_2_ differences can introduce a pronounced bias, which may obscure the gagCEST effect when using low duty cycles and long saturation trains [134].

*Diffusion tensor imaging* (DTI) requires sufficient spatial resolution for depiction of cartilage with relatively short T_2_ and is thus limited by low SNR. Raya et al. used the greater SNR at 7T in combination with a line scan DTI sequence to show decreased fractional anisotropy (FA) and increased ADC in knee cartilage of OA patients compared to healthy volunteers [16].

**Fig. 12:**
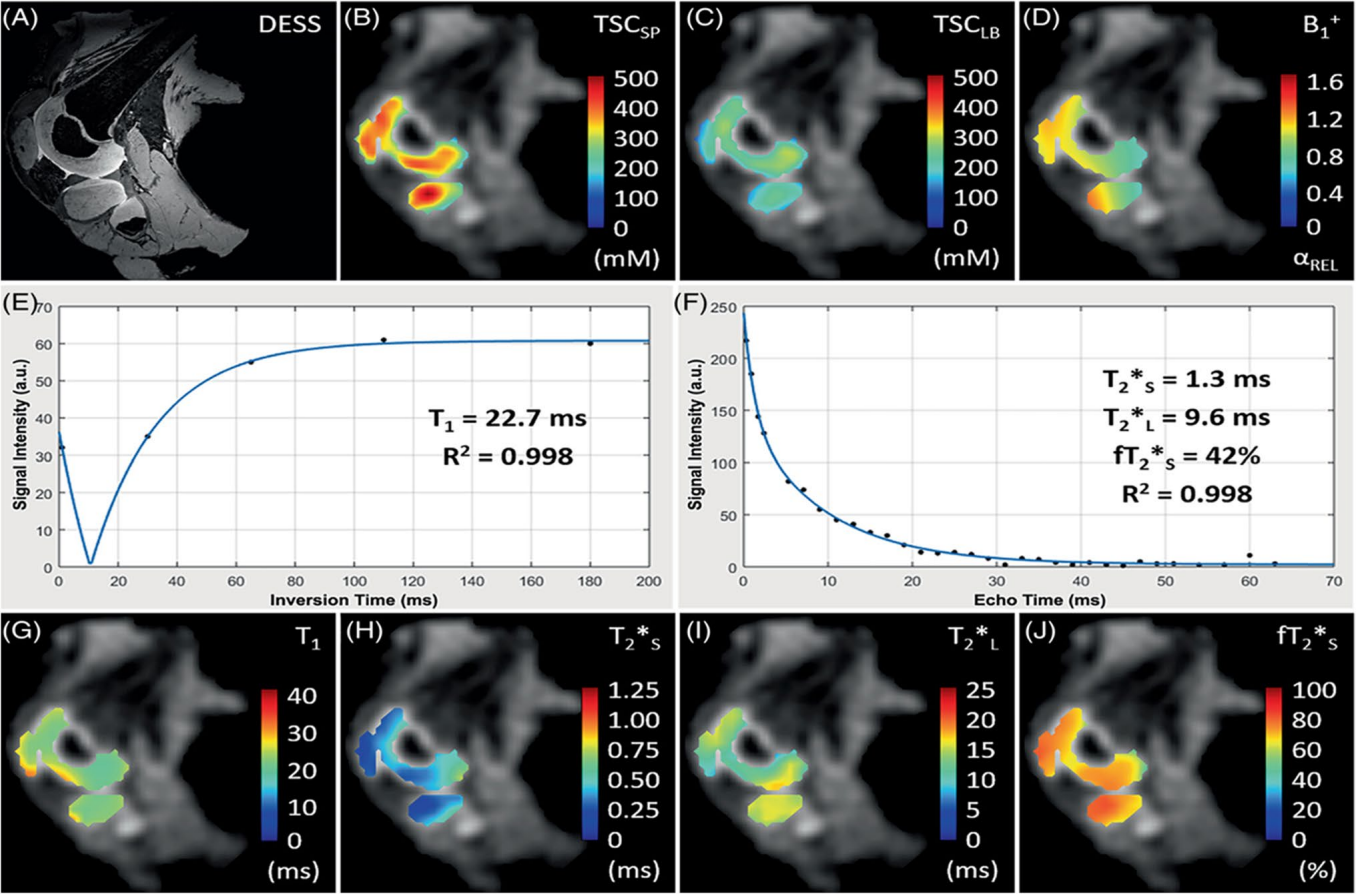
Ex vivo sodium MRI from the right knee of a 4.5‐year‐old male donor at 10.5 Tesla. **A** High‐resolution proton 3D DESS image showing cartilage in the lateral femoral condyle, tibia, patella, and fibula. **B** and **C** Color‐coded quantitative sodium (^23^Na) maps overlaid on the ^23^Na density images from the same location as the 3D DESS image. The TSC_SP_ map corrected for specimen‐specific cartilage parameters (**B**) showed higher concentrations compared to the TSC_LB_ map corrected using literature‐based cartilage parameters (**C**). **D** The B_1_ + map. **E** Example fits (blue) of data points (black dots) from a single pixel acquired using an inversion recovery experiment for T1 mapping. **F** Multi‐echo experiment for biexponential T2* fitting. **G–J** Relaxation maps showing the distribution of T_1_(G), T_2_*s (H), T_2_*l (I), and fT_2_*s (J) in femoral, tibial, and patellar cartilage regions. DESS, double echo steady‐state; TSC, tissue sodium concentration; SP, specimen‐specific; LB, literature‐based; T_2_*s: short T_2_* component; T_2_*l: long T_2_* component; fT_2_*s signal fraction relaxing with T_2_*s values. Figure from Reference 132 with permission

In *sodium (*^*23*^*Na) MRI*, direct imaging of ^23^Na with MRI is difficult compared to standard proton MRI due to low tissue concentrations, inherently low SNR, very short T_2_s, and very low MR sensitivity (only 9.3% of proton MR). 7T can greatly improve ^23^Na MRI due to the increased SNR. Madelin et al. demonstrated ^23^Na MRI was sensitive to changes in cartilage GAG content in OA patient knees over only 16 months [135]. ^23^Na MRI was also used to compare the quality of cartilage repair tissue 33 months after two different surgical approaches [136]. A recent ex vivo 10.5T study of human pediatric knee specimens by Zbyn et al. demonstrated that ^23^Na concentration and ^23^Na relaxation times can non-destructively follow changes in sGAG content and collagen matrix during cartilage maturation (Fig. 12) [137].

## Current gaps and future directions

The past three decades have seen substantial advancements in Cart-C MRI. However, an important question remains: why, after 30 years of development, is Cart-C MRI not yet widely adopted in clinical practice or as a primarily outcome measures for large scale clinical trials?

Link et al. previously laid out a number of essential elements that are required to apply Cart-C MRI clinically and in clinical trials [138]: (i) fast (accelerated) and reproducible imaging techniques, (ii) automated cartilage segmentation and analysis techniques, (iii) standardized methodology that is reproducible and uniform across MRI scanners irrespective of the vendor, and (iv) reference values with a definition of normal and abnormal values. Over the past decade, significant technical advancements have been made for the first two elements, i.e., accelerated acquisition and automated analysis, particularly with the aid of deep learning-based techniques. For example, the deep learning reconstruction superMAP allows simultaneously collect cartilage T_1ρ_ and T_2_ maps of the whole knee within 2 min [66]; the full OAI dataset has been analyzed automatically for T_2_ values [88]. However, the automated analysis methods have been primarily limited to homogeneous research cohort data such as OAI. Developing deep learning models that are generalizable and thus clinically useful is challenging since these images vary greatly from scanner to scanner and site to site. Furthermore, the image processing pipeline needs to be integrated into the clinical imaging workflow seamlessly before clinical implementation. For accelerated image acquisition, previous studies have been primarily limited to retrospective undersampling. More validation studies with a larger sample size of prospective undersampling and across different MR platforms are warranted before these techniques can be translated into clinical use.

Regarding standardization of the methodology across different MRI scanners, the MSK Quantitative Imaging Biomarker Committee (QIBC, formerly MSK committee under the Radiological Society Radiological Society of North America (RSNA)/Quantitative Imaging Biomarker Alliance [QIBA]) has assembled an expert team of radiologists, imaging researchers, clinicians (e.g., orthopedic surgeons, rheumatologists) from more than 40 institutions and involved industrial partners. The taskforce has provided recommendations pertaining to image data acquisition, analysis, and interpretation and assessment procedures for T_1ρ_ and T_2_ cartilage imaging and test–retest conformance [139]. This is a timely effort; ccMRI has demonstrated good to excellent reproducibility in single site or in multi-site single-vendor studies [39]; however, few studies have examined its reliability in a multi-site, multi-vendor context. Such reliability is crucial for its applications in clinical practice and in large-scale multi-site multi-vendor trials. One study reported that the inter-vendor mean T_2_ differences ranged 5.4 to 10.0 ms (10 ~ 25%) using vendor product T_2_ imaging sequences [140]. Two recent studies showed that inter-vendor variations of T_1ρ_ and T_2_ values can be reduced to approximately 10% through more harmonized sequence design and protocol setup, highlighting the importance of standardizing data acquisition [50, 141].

**Fig. 13:**
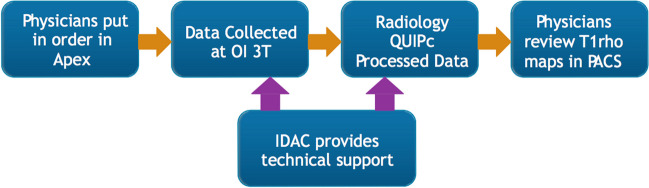
Workflow for integrating T_1ρ_ imaging acquisition and processing into clinical operations. Physicians ordered T_1ρ_ imaging through the standard ordering system (APEX). The data were collected by clinical technologists using the 3 Tesla MR scanner at the UCSF Orthopaedic Institute (OI) imaging center. T_1ρ_ images were automatically transferred to the clinical PACS system as all other clinical MR images. Cartilage segmentation was performed by the UCSF Radiology Quantitative Image Processing (QUIPc) group within 1–2 days after data collection. Color maps of T_1ρ_ within the segmented cartilage were generated and pushed back to the clinical PACS system immediately after image analysis, which allowed physicians to review T_1ρ_ maps directly from the clinical PACS. Technical support for both data acquisition and processing was provided by the Imaging and Data Analysis Core (IDAC) within the Center of Research Translation of the Study of Osteoarthritis at UCSF and UC-Davis (NIH/NIAMS P50AR060752)

**Fig. 14:**
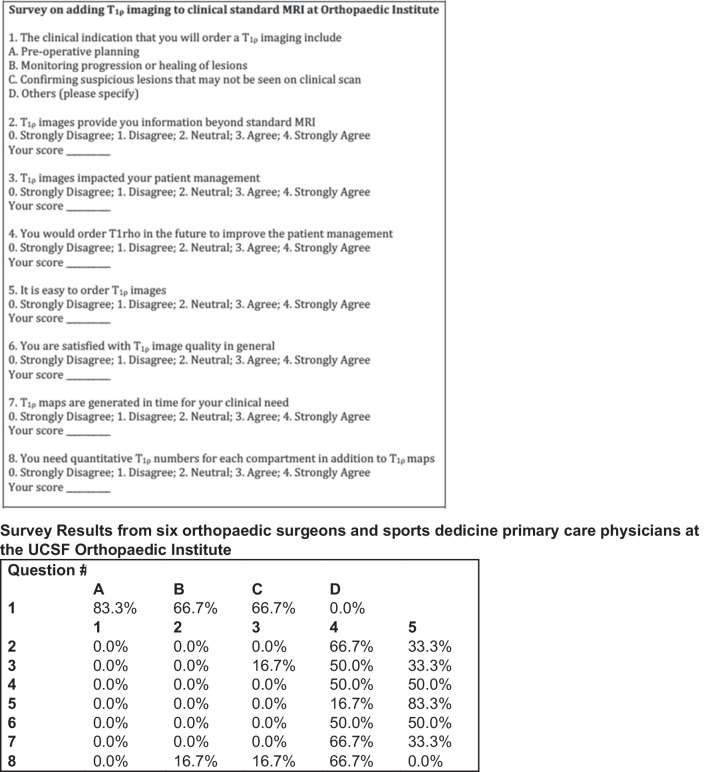
Survey questions (top) and results (bottom) on adding T_1ρ_ imaging to standard clinical MRI

**Fig. 15:**
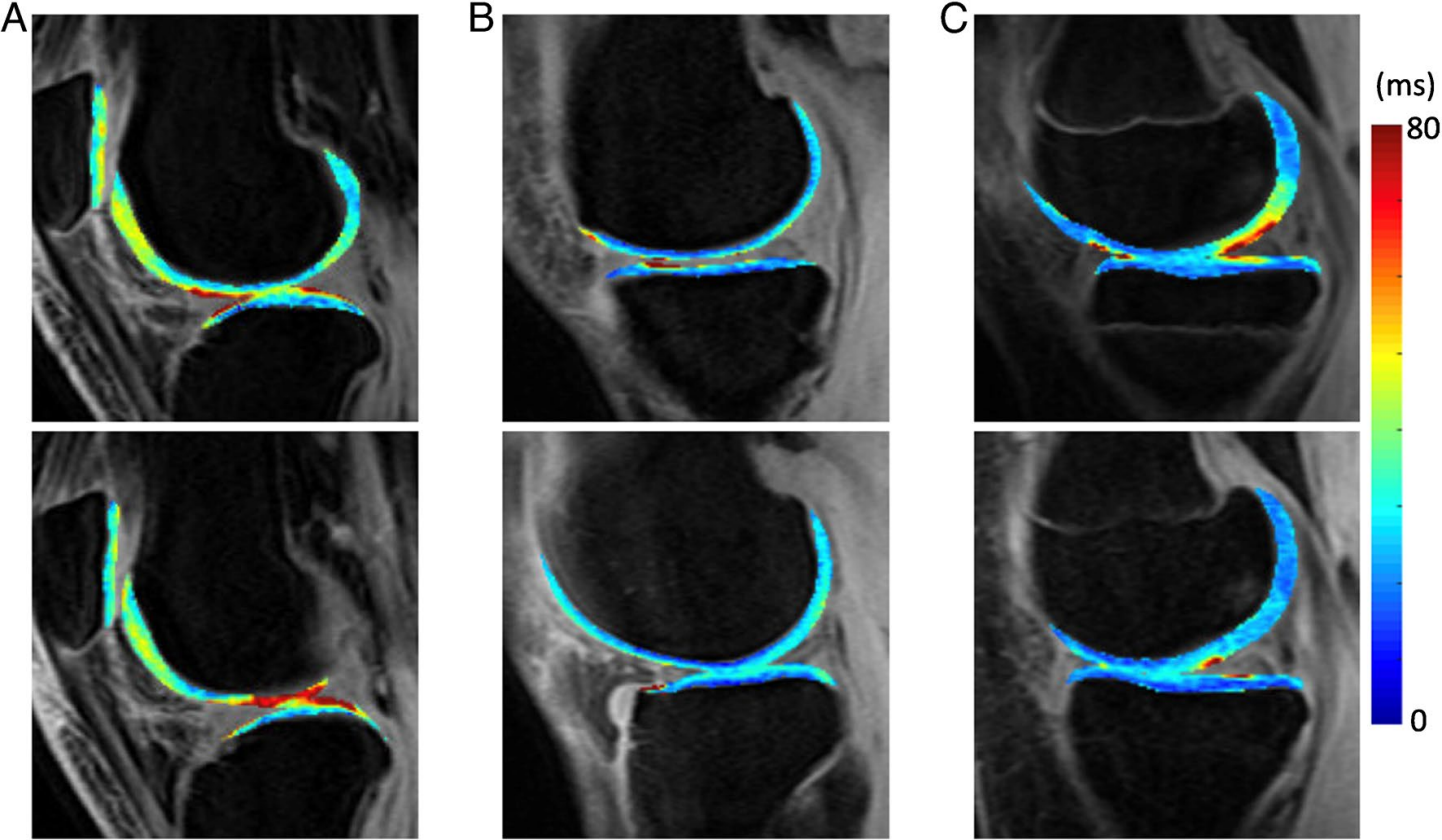
Clinical utility of ccMRI. **A** Cartilage T_1ρ_ maps of a 23-year-old female obtained in August 2012 (top) and June 2013 (bottom). T_1ρ_ maps in June 2013 showed progressive cartilage damage in lateral femoral condyle and lateral tibia (indicated as red regions). The patient had lateral meniscal deficiency and underwent meniscus transplant in July 2013, 1 month after the bottom images were collected. **B** A 49-year-old male had medial cartilage damage/degeneration and meniscal tear. T_1ρ_ imaging (top) showing early medial compartment cartilage changes while the lateral compartment cartilage (bottom) was healthy, justifying a high tibial-osteotomy (HTO). **C** A 14-year-old female following a lateral meniscectomy and chondroplasty due to meniscal injury and partial thickness cartilage defects. T_1ρ_ imaging was performed in Sept 2015 (top) and Feb 2017 (bottom), showing improvement of cartilage health in medial femoral condyle and medial tibia after the surgery

Another fundamental question is: what additional clinical value can Cart-C MRI offer as compared to the current clinical MRI? Kijowski et al. added T_2_ mapping to a routine MR protocol at 3T in 150 patients and demonstrated significantly improved sensitivity in detecting cartilage lesions with the addition of T_2_ mapping [142]. Beyond diagnostic utility, the potential of Cart-C MRI to enhance patient management also warrants evaluation. At the Orthopaedic Institute, University of California San Francisco (UCSF), cartilage T_1ρ_ mapping was integrated into the clinical routine for 390 patients (278 in knees and 112 in hips) between 2011 and 2017. A workflow was established that allowed the referring physicians to review T_1ρ_ maps directly from the clinical PACS (Fig. 13). A survey was conducted among six referring clinicians to evaluate the potential clinical value of T_1ρ_ imaging (Fig. 14). The primary indications for ordering T_1ρ_ mapping included preoperative planning, monitoring progression or healing of lesions, or confirming suspicious lesions that may not be seen on clinical scans. The clinicians were satisfied with the T_1ρ_ image quality in general, and all clinicians unanimously indicated they would like to order T_1ρ_ imaging to improve their patient management in the future (50% agree and 50% strongly agree). Figure 15 showed three cases where T_1ρ_ mapping provided useful information to clinicians for patient management. These studies offer compelling evidence that Cart-C MRI can contribute additional clinical value for both diagnosis and patient management.

Another key barrier to the clinical adoption of Cart-C MRI and other quantitative MRI techniques stems from the paucity of OA therapies and lack of any approved disease modifying OA drugs (DMOAD). Upon the approval of a DMOAD, cartilage quantitative MRI techniques should become clinically necessary for patient selection and evaluating treatment response and disease progression. Ongoing efforts to develop rapid acquisition techniques, integrate automated analysis into clinical flow, standardize acquisition and analysis protocols, and establish reference values are essential steps in preparing for this transition.

## Conclusions

The past three decades have produced significant technical developments for cartilage evaluation. Cart-C MRI can detect early cartilage degeneration by probing changes within the collagen-PG matrix. Numerous studies have shown the potential of Cart-C MRI to serve as diagnostic, prognostic, and predictive biomarkers for diseases and injuries that impact cartilage. However, further steps are needed to bring these advanced imaging techniques into routine clinical practice and clinical trials. Further validation of accelerated imaging acquisition and automated analysis techniques, seamless integration into clinical workflows, and standardization the techniques across different MR systems will be required. With further implementation in these areas, Cart-C MRI can help to enhance preoperative planning, and monitoring progression or healing of lesions in clinical practice. In parallel, broader applications of compositional MRI in large-scale clinical trials is essential for generating sufficient data for biomarker qualification and successful DMOAD trials. The discovery and approval of successful DMOADs would undoubtedly elevate the clinical applications of Cart-C MRI. In this “chicken-egg” dilemma, synergized efforts between researchers, clinicians, and industrial partners are essential to advance the field.
